# Clinical Outcomes of Eravacycline in Patients Treated Predominately for Carbapenem-Resistant Acinetobacter baumannii

**DOI:** 10.1128/spectrum.00479-22

**Published:** 2022-10-03

**Authors:** Sara Alosaimy, Taylor Morrisette, Abdalhamid M. Lagnf, Leonor M. Rojas, Madeline A. King, Benjamin M. Pullinger, Athena L. V. Hobbs, Nicholson B. Perkins, Michael P. Veve, Jeannette Bouchard, Tristan Gore, Bruce Jones, James Truong, Justin Andrade, Glen Huang, Reese Cosimi, S. Lena Kang-Birken, Kyle C. Molina, Mark Biagi, Michael Pierce, Marco R. Scipione, Jing J. Zhao, Susan L. Davis, Michael J. Rybak

**Affiliations:** a Anti-Infective Research Laboratory, Department of Pharmacy Practice, Eugene Applebaum College of Pharmacy and Health Sciences, Wayne State University, Detroit, Michigan, USA; b Valley Hospital Medical Center, Las Vegas, Nevada, USA; c Philadelphia College of Pharmacy, University of the Sciences, Philadelphia, Pennsylvania, USA; d Cooper University Hospital, Camden, New Jersey, USA; e Methodist University Hospital, Memphis, Tennessee, USA; f Cardinal Health, Memphis, Tennessee, USA; g University of Tennessee Health Science Center College of Pharmacy, Memphis, Tennessee, USA; h University of Tennessee Medical Center, Knoxville, Tennessee, USA; i Department of Pharmacy, Henry Ford Hospital, Detroit, Michigan, USA; j College of Pharmacy, University of South Carolina, Columbia, South Carolina, USA; k St. Joseph’s/Candler Health System, Savannah, Georgia, USA; l The Brooklyn Hospital, Brooklyn, New York, USA; m University of California Los Angeles, Los Angeles, California, USA; n Ascension St. Vincent Hospital, Indianapolis, Indiana, USA; o Santa Barbara Cottage Hospital, Santa Barbara, California, USA; p University of Colorado Skaggs School of Pharmacy and Pharmaceutical Sciences, Aurora, Colorado, USA; q SwedishAmerican Hospital, Rockford, Illinois, USA; r University of Illinois at Chicago, Rockford, Illinois, USA; s Department of Pharmacy, Detroit Receiving Hospital, Detroit Medical Center, Detroit, Michigan, USA; t Department of Pharmacy, Harper University Hospital, Detroit Medical Center, Detroit, Michigan, USA; u Department of Medicine, Division of Infectious Diseases, School of Medicine, Wayne State University, Detroit, Michigan, USA; Emory University School of Medicine

**Keywords:** eravacycline, *Acinetobacter baumannii*, *Acinetobacter*, CRAB, Gram-negative bacteria, hospital infections, tetracyclines

## Abstract

Forty-six patients were treated with eravacycline (ERV) for Acinetobacter baumannii infections, where 69.5% of isolates were carbapenem resistant (CRAB). Infections were primarily pulmonary (58.3%), and most patients received combination therapy (84.4%). The median (IQR) ERV duration was 6.9 days (5.1 to 11.1). Thirty-day mortality was 23.9% in the cohort and 21.9% in CRAB patients. One patient experienced an ERV-possible adverse event.

**IMPORTANCE** Acinetobacter baumannii, particularly when carbapenem resistant (CRAB), is one of the most challenging pathogens in the health care setting. This is complicated by the fact that there is no consensus guideline regarding management of A. baumannii infections. However, the recent Infectious Diseases Society of America guidelines for treatment of resistant Gram-negative infections provided expert recommendations for CRAB management. The panel suggest using minocycline among tetracycline derivatives rather than eravacycline (ERV) until sufficient clinical data are available. Therefore, we present the largest multicenter real-world cohort in patients treated with ERV for A. baumannii, where the majority of isolates were CRAB (69.5%). Our analysis demonstrate that patients treated with ERV-based regimens achieved a 30-day mortality of 23.9% and had a low incidence of ERV-possible adverse events (2.1%). This study is important as it fills the gap in the literature regarding the use of a novel tetracycline (i.e., ERV) in the treatment of this challenging health care infection.

## INTRODUCTION

Acinetobacter baumannii is one of the most challenging pathogens in the health care setting (1, 2). The attributable mortality in A. baumannii ventilator-associated pneumonia and bloodstream infections is as high as 54% in the intensive care unit. In fact, it is associated with the highest attributable costs compared to various resistant pathogens in both community onset and hospital-onset invasive infections—up to $128,235 (3). Furthermore, these isolates are often resistant to a wide number of antibiotic classes, such as fluoroquinolones and polymyxins, and have the ability to rapidly acquire resistance to many agents, such as carbapenem. Notably, carbapenem-resistant A. baumannii (CRAB) is considered to be an urgent threat according to the most recent report from the Centers of Disease Control and Prevention (CDC), due to a lack of available therapeutic options (4). There is no current consensus regarding a definition of treatment success for A. baumannii infections (5, 6). Regarding CRAB management, however, the recent Infectious Diseases Society of America guidelines for treatment of resistant Gram-negative infections provided expert recommendations (7). The panel recommended treatment with a single agent, preferably ampicillin-sulbactam, for mild CRAB infections. For moderate to severe CRAB, combination therapy with at least 2 agents, preferably with *in vitro* activity, is suggested. Regarding the use of tetracycline (TET) derivatives in CRAB, the panel suggest using minocycline or even high-dose tigecycline as alternative rather than eravacycline (ERV) until more clinical data with ERV in CRAB become available.

ERV is a novel fluorescein within the tetracycline (TET) class approved by the Food and Drug Administration (FDA) for the treatment of complicated intra-abdominal infections (8). Unlike real-world studies, clinical trials thus far have exclusively focused on the infection site rather than the causative organism and are conducted under ideal conditions in patient populations that are different from the daily clinical practice patient. In fact, although existing clinical trials and/or real-world reports of ERV have been slowly emerging, only 15 patients with A. baumannii were included (9–11). While these reports have described positive clinical outcomes, the majority of these A. baumannii cases were not CRAB and/or their carbapenem susceptibilities were not indicated. The objective of this study is to describe real-world experience with ERV for the treatment of A. baumannii, including CRAB, to evaluate its effectiveness and adverse events.

## RESULTS

Collectively, 46 patients who received ERV for A. baumannii were included (Table 1). The median (interquartile range [IQR]) age was 62.5 years (52.5 to 76.0 years), and the majority of patients were male (54.3%) and Caucasian (54.3%). The median (IQR) APACHE-II score was 15 (11 to 24), and many patients were in the intensive care unit at time of index culture (41.3%). Medical comorbidities were common: primarily obesity (37.0%), diabetes (37.0%), chronic pulmonary disease (30.4%), cerebrovascular disease (32.6%), and chronic kidney disease (23.9%). The majority of patients (91.3%) had at least one risk factor for multidrug-resistant infections: the most common was the use of antimicrobials for ≥24 h in the 90 days before index culture (67.4%), followed by hospitalization for ≥48 h in the 90 days before index culture 63.0%.

**TABLE 1:** Patient demographics, clinical criteria and health outcomes^*a*^

Parameter	Result for^*b*^:	
	Population (*n* = 46)	CRAB (*n* = 32)
Age, yr, median (IQR)	62.5 (52.5–76)	62.0 (53.0–77.0)
Age of ≥65 yr, *n* (%)	20 (43.5)	15 (46.9)
Sex, male, *n* (%)	25 (54.3)	17 (53.1)
Race, *n* (%)		
African-American	13 (28.3)	7 (21.9)
Caucasian	25 (54.3)	20 (62.5)
Other	8 (17.3)	5 (15.6)
wt, kg, median (IQR) BMI, kg/m^2^, median (IQR)	71.6 (60.2–91.2) 25.1 (22.0–33.5)	79.3 (61.8–92.1) 25.7 (21.9–34.9)
BMI of ≥30	17 (37.0%)	13 (40.6%)
Baseline serum creatinine, mg/dL, median (IQR)	0.80 (0.6–1.5)	0.85 (0.6–1.4)
CL_CR_, mL/min, median (IQR)	18.6 (14.7–35.5)	19.0 (13.2–38.9)
Residence prior to admission, *n* (%)		
Home	19 (41.3)	11 (34.4)
Transfer from outside hospital	5 (10.9)	5 (15.6)
Nursing home, skilled nursing facility, long term care facility	18 (39.1)	16 (50.0)
Other^*c*^	4 (91.3)	0 (0)
Comorbid conditions		
CCI, median (IQR)	4.0 (2.0–7.0)	4.5 (2.0–6.8)
CCI of ≥5, *n* (%)	21 (45.7)	16 (50.0)
Cerebrovascular disease, *n* (%)^*d*^	15 (32.6)	12 (37.5)
Chronic pulmonary disease, *n* (%)^*e*^	14 (30.4)	12 (37.5)
Moderate to severe kidney disease or on chronic dialysis, *n* (%)^*f*^	11 (23.9)	9 (28.1)
Connective tissue disease, *n* (%)^*g*^	3 (6.5)	0 (0)
Clostridiodes difficile-associated diarrhea, *n* (%)	3 (6.5)	3 (9.4)
Dementia, *n* (%)	8 (17.4)	4 (12.5)
Diabetes, *n* (%)	17 (37.0)	11 (34.4)
Heart failure, *n* (%)	11 (23.9)	7 (21.9)
Hemiplegia, *n* (%)	6 (13.0)	5 (15.6)
Tumor without metastasis, *n* (%)	3 (6.5)	3 (9.4)
Tumor with metastasis, *n* (%)	4 (8.7)	1 (3.1)
Liver disease, *n* (%)	1 (2.2)	1 (3.1)
Myocardial infarction, *n* (%)	2 (4.3)	2 (6.3)
No other conditions, *n* (%)	6 (13.0)	5 (15.6)
Peptic ulcer disease, *n* (%)	4 (8.7)	3 (9.4)
Peripheral vascular disease, *n* (%)^*h*^	3 (6.5)	3 (9.4)
PWID, *n* (%)	3 6.5)	2 (6.3)
MDR risk factors, *n* (%)^*i*^		
Admitted from nursing home or extended care facility	22 (47.8)	19 (59.4)
Chronic dialysis in 30 days before index culture	6 (13.0)	5 (15.6)
Colonization with resistant organisms	15 (32.6)	11 (34.4)
Home infusion therapy	3 (6.5)	1 (3.1)
Home wound care	5 (10.9)	3 (9.4)
Prior antimicrobials for >24 h in 90 days prior to index culture	31 (67.4)	21 (65.6)
Prior infection with resistant organisms at any time	22 (47.8)	14 (43.8)
Prior hospitalization for at least 48 h in 90 days prior to index culture	29 (63.0)	21 (65.6)
Prior surgery in 30 days preceding index culture	7 (15.2)	6 (18.8%)
Sources of infection, *n* (%)		
Bone and joint	4 (8.7)	3, (9.4)
Intra-abdominal	2 (4.3)	2, (6.3)
Primary bacteremia	3 (6.5)	1, (3.1)
Pneumonia	24 (52.2)	19, (59.4)
Mechanically ventilated for 48 h prior to pneumonia^*j*^	18 (75.0)	14, (73.7)
Skin and soft tissue	8 (17.4)	5 (15.6)
Urinary	2 (4.3)	0 (0)
Unknown	1 (2.2)	0 (0)
Other^*k*^	2 (4.3)	2 (6.3)
Pathogens targeted beyond A. baumannii, *n* (%)		
Enterobacter cloacae	1 (2.2)	1 (3.1)
Enterococcus faecalis	2 (4.3)	1 (3.1)
Enterococcus faecium	4 (8.7)	2 (6.3)
Escherichia coli	3 (6.5)	3 (9.4)
Klebsiella oxytoca	1 (2.2)	1 (3.1)
Klebsiella pneumoniae	4 (8.7)	2 (6.3)
Morganella morganii	1 (2.2)	1 (3.1)
Proteus mirabilis	1 (2.2)	1 (3.1)
Providencia stuartii	1 (2.2)	1 (3.1)
Staphylococcus aureus	7 (15.2)	3 (9.4)
Methicillin resistant	4 (8.7)	3 (9.4)
Stenotrophomonas maltophilia	1 (2.2)	1 (3.1)
Other^*l*^	6 (13.0)	6 (18.8)
MIC		
Ampicillin-sulbactam (*n* = 35), mg/L, median (range)	16.0 (2.0–32.0)	16.0 (4.0–32.0)
Susceptible, *n* (%)	8/35 (22.9)	4/27 (14.8)
Amikacin (*n* = 21), mg/L, median (range)	32.0 (4.0–256.0)	32.0 (10.0–32.0)
Susceptible, *n* (%)	11/21 (52.4)	8/20 (40.0)
Ceftazidime-avibactam (*n* = 1), mg/L	128	128
Colistin (*n* = 2), mg/L, median (range)	1.5 (1.0–2.0)	1.5 (1.0–2.0)
Intermediate, *n* (%)^*m*^	2/2 (100)	2/2 (100)
Eravacycline (*n* = 6), mg/L, median (range)^*n*^	0.87 (0.125–1.50)	0.75 (0.5–1.0)
Imipenem (*n* = 9), mg/L, median (range)	16.0 (0.25–16.0)	16.0 (8.0–16.0)
Susceptible, *n* (%)	1/9 (11.11)	0/8 (0)
Tigecycline (*n* = 17), mg/L, median (range)	2.0 (0.5–4.0)	2.0 (1.0–4.0)
Tobramycin (*n* = 38), mg/L, median (range)	4.0 (1.0–16.0)	8.0 (1.0–16.0)
Susceptible, *n* (%)	20/38 (52.6)	13/29 (44.8)
Meropenem (*n* = 37), mg/L, median (range)	8.0 (0.25–16)	16.0 (8–16)
Susceptible, *n* (%)	5/37 (13.5)	0/32 (0)
Meropenem-vaborbactam (*n* = 1), mg/L	64.0	64.0
Minocycline (*n* = 17), mg/L, median (range)	8.0 (1.0–32.0)	8.0 (2.0–32.0)
Susceptible	6/17 (35.3)	5/14 (35.7)
Markers of disease progression		
APACHE II, median (IQR)	15.0 (11.0–24.0)	17.5 (10.3–24.8)
APACHE score of ≥15, *n* (%)	26 (56.5)	19 (59.4)
APACHE score of ≥30, *n* (%)	3 (9.4)	4 (8.7)
ERV		
Duration, days, median (IQR)	6.9 (5.1–11.1)	7.5 (5.4–11.9)
Start from index culture, days, median (IQR)	3.0 (2.0–5.7)	3.4 (2.3–5.5)
Dosing, *n* (%)		
1 mg/kg q12h	44 (95.7)	32 (100)
Other^*o*^	2 (4.3)	0 (0)
Treatment-related factors		
Active antibiotics prior to ERV, *n* (%)^*p*^	16 (34.8)	11 (34.4)
Inhaled antibiotics, any, *n* (%)^*q*^	12 (26.1)	10 (31.3)
i.v. combination therapy for ≥48 h, *n* (%)	39 (84.8)	24 (75.0)
Amikacin	4 (8.7)	3 (9.4)
Ampicillin-sulbactam	6 (13.0)	6 (18.8)
Colistin	4 (8.7)	4 (12.5)
Meropenem	8 (17.4)	0 (0)
Polymyxin B	4 (8.7)	4 (12.5)
Other^*r*^	13 (33.3)	7 (21.8)
Intensive care upon index culture, *n* (%)^*s*^	19 (41.3)	14 (43.8)
SOFA score, median (IQR)	4.0 (2.0–7.0)	5.0 (2.3–7.0)
Mechanical ventilation, *n* (%)	18 (39.1)	14 (43.8)
For ≥48 h	18 (100)	14 (100)
Surgery consult, *n* (%)	17 (37.0)	13 (40.6)
Source control, *n* (%)^*t*^	20 (43.5)	16 (50)
Infectious Diseases consult, *n* (%)	45 (97.8)	31 (96.9)
Within 48 h^*u*^	34/45 (75.5)	25/31 (80.6)
Switched to another agent, *n* (%)	6 (13.0)	3 (9.4)
Minocycline	3 (6.5)	1 (3.1)
Other^*v*^	3 (6.5)	2 (6.2)
Clinical outcomes		
30-day mortality, *n* (%)	11 (23.9)	7 (21.9)
90-day mortality, *n* (%)	14 (30.4)	10 (31.3)
30-day recurrence, *n* (%)	10 (21.7)	8 (25.0)
Excluding patients with 30-day mortality	7 (20.0)	6 (24.0)
30-day readmission, *n* (%)	7 (15.2)	5 (15.6)
Excluding patients with 30-day mortality	6 (17.1)	4 (16.0)
Symptoms of infection worsen or fail to resolve, *n* (%)	13 (28.3)	9 (28.1)
Excluding patients with 30-day mortality	7 (20.0)	4 (16.0)
LOS, median (IQR)		
Total	21 (12.5–39.0)	22 (13.0–39.5)
Before index culture	13.6 (10.1–30.9)	14.3 (10.5–31.5)
ICU	23.0 (16.5–46.5)	23.5 (16.5–47.0)
ERV-possible adverse events, *n* (%)		
Gastrointestinal	1 (2.2)	1 (3.1)

a APACHE II, Acute Physiology and Chronic Health Evaluation; BMI, body mass index; CCI, Carlson comorbidity index; CL_CR_, creatinine clearance; CRAB, carbapenem-resistant Acinetobacter baumannii; CD4, cluster of differentiation 4; COPD, chronic obstructive pulmonary disease; DVT, deep venous thrombosis; HIV, human immunodeficiency virus; LOS, length of stay; MDR, multidrug resistant; MEV, meropenem-vaborbactam; OA, osteoarthritis; PWID, person who injects drugs; q12h, every 12 h; SOFA, sequential organ failure assessment; TIA, transient ischemic attack.

b Data are presented as median (IQR) and/or *n* (percentage) as appropriate. MIC data are presented as median (range).

c Long-term acute care facility (*n* = 2) or unknown (*n* = 2).

d Stroke or TIA.

e Asthma or COPD.

f Hemodialysis or peritoneal dialysis.

g Osteoarthritis or rheumatic arthritis.

h Deep venous thrombosis, chronic venous disease.

i Defined as nonsusceptible to ≥3 antimicrobial categories.

j Suggestive of ventilator-associated pneumonia.

k Sacral wound (*n* = 1) and infected peritoneal dialysis site (*n* = 1).

l Enterobacter cloacae, Klebsiella oxytoca, Morganella morganii, Proteus mirabilis, Providencia stuartii, Stenotrophomonas maltophilia (*n* = 1 each).

m The CLSI has assigned only an intermediate or resistant interpretation for colistin activity: MICs of ≤2 mg/L and >2 mg/L for each designation, respectively.

n *n* = 6 used Etest for ERV MIC in three institutions. A fourth institution used the Kirby-Bauer disk diffusion susceptibility test (*n* = 2), where the zones of inhibition were measured at 12 and 14 mm, respectively.

o *n* = 1 received 0.78 mg per kg q12h (to round down to 100 mg), *n* = 1 received 4 doses of 1 mg/kg q12h and then 1.1 mg/kg (to round up to 75 mg).

p Active therapy before ERV was defined as therapy deemed to have *in vitro* susceptibility for A. baumannii administered before ERV start date.

q Colistin (*n* = 6), tobramycin (*n* = 4), and amikacin (*n* = 1).

r Cefepime, cefiderocol, ceftriaxone, and tobramycin (*n* = 3 each) and ciprofloxacin (*n* = 1).

s Seventeen (89.5%) medical, 1 (5.3%) cardiac, and 1 (5.3%) surgical/trauma.

t Debridement (*n* = 7), incision and drainage (*n* = 3), intravenous catheter removal (*n* = 1), invasive device removal (*n* = 1), amputation (*n* = 1), other (*n* = 11).

u From index culture. If ID was consulted before, index culture time was considered to be within 0 h.

v Ampicillin-sulbactam (*n* = 2), meropenem (*n* = 1).

The majority of patients had an Infectious Diseases (ID) consultation (97.8%), and a significant proportion of patients were consulted for ID prior to the positive index culture (37.2%). Among these consulted after positive culture (*n* = 27), the median (IQR) time to ID consult was 37.6 h (1.5 to 80.6 h). Surgical interventions related to the infection source were established in 37.0%. The sources of infection were variable, but pulmonary infection (52.2%) was the most common, followed by skin/soft tissue (17.4%) and bone/joint (8.7%) infections. A. baumannii was most commonly isolated from expectorated sputum (37.0%), blood (17.4%), wound/tissue (17.4%), or bronchoalveolar lavage fluid (6.5%). Among patients with microbiological clearance (*n* = 10), the median (IQR) time to clear was 1.9 days (1.6 to 5.8 days). The sources of infection for patients who cleared were mostly respiratory tract (4/10), followed by skin, bone/joint, primary bacteremia, and intra-abdominal (*n* = 1, each). Other pathogens were also isolated, mainly Staphylococcus aureus (15.2%), followed by Enterococcus faecium and Klebsiella pneumoniae (8.7% each).

The majority of patients received combination therapy (84.8%), with meropenem being the most commonly used second agent (17.4%). When ERV was switched to another agent (13.0%), minocycline was most frequently selected (6.5%).

Thirty-day mortality occurred in 23.9% of the entire cohort and 21.9% of patients with CRAB. Thirty-day recurrence occurred in 21.7% and 25.0% of patients in the entire cohort and CRAB cohort, respectively. Table 1 provides a detailed list of clinical criteria and outcomes of patients for the entire cohort versus the CRAB cohort.

MIC values for ERV were tested using the Etest in six isolates from three institutions, the median of which was 0.87 mg/L (range, 0.125 to 1.50 mg/L). In a fourth institution, ERV susceptibility was evaluated using Kirby-Bauer disk diffusion for two isolates, of which the median was 13.00 mm (range, 12 to 14 mm). On the other hand, MIC values for other commonly utilized antimicrobials were available for all isolates, primarily for meropenem (80.4%), ampicillin-sulbactam (76.0%), and amikacin (76.0%).

ERV was generally initiated early at a median (IQR) of 3.0 days (2.0 to 5.7 days) from the index culture. All except two patients received the full recommended dose of ERV. The total median duration of ERV was 6.9 days (5.1 to 11.1 days). Most patients received ≥2 combination intravenous (i.v.) antibiotics (84.8%), with the most common being meropenem (17.4%), ampicillin-sulbactam (13.0%), and amikacin and colistin (8.7%, each).

Only one patient experienced an ERV-possible adverse event (gastrointestinal), which was diarrhea grade I while on therapy, which occurred 10 days after initiation of ERV. Nevertheless, this did not lead to drug discontinuation, and the patient did not experience mortality, recurrence, readmission, or worsening of symptoms.

## DISCUSSION

In this brief report, we present the largest real-world, observational analysis of treatment outcomes for patients treated with ERV for an A. baumannii infection, which were predominantly carbapenem resistant. *In vitro* studies report ERV MIC_90_ ranges of 0.5 to 2 and 1 to 2 mg/L in A. baumannii and CRAB, respectively (8). The IGNITE1 trial (ClinicalTrials registration no. NCT01844856). showed clinical cure with ERV in 8/8 A. baumannii patients, of which 2/2 were confirmed carbapenemase producers (9). Similarly, the IGNITE4 trial (ClinicalTrials registration no. NCT01844856) demonstrated an ERV success rate (as prespecified by the FDA) in 5/5 A. baumannii patients with complicated intra-abdominal infection (cIAI) (10). Additionally, the IGNITE1 and IGNITE4 trials were limited to a specific infection site (i.e., cIAI) rather than other sites with more common A. baumannii infections, such as pulmonary, blood, and urine (12). We recently reported our early experience with ERV in the real-world setting for various infection types, where 5/7 patients treated for A. baumannii experienced 30-day survival (11). Collectively, the majority of patients described in the literature either were not CRAB or carbapenem susceptibilities were not reported. It is challenging to extrapolate the results of the few published postmarketing clinical experiences to A. baumannii patients seen in the real-world clinical setting.

In this cohort, we reviewed outcomes of patients with A. baumannii treated with ERV for various infections, including cIAI, and with nearly 70% of isolates being CRAB. Compared to other historical treatment options for CRAB, such as colistin and the newer cephalosporin agents, such as cefiderocol, ERV 30-day survival was promising at nearly 75% (12, 13). When ERV was switched to another agent, minocycline was the most common agent selected, probably due to oral availability.

Nearly 85% of patients in our cohort received combination antibiotics with ERV as observed in most A. baumannii reports, particularly when patients present with CRAB. That was not surprising because combination approaches that include a carbapenem, polymyxin B, and/or ampicillin-sulbactam are often advocated for vulnerable and critically ill patients infected with CRAB (14). Albeit evidence is not optimum, the use of combination therapy is hypothesized to increase the likelihood of adequate empirical antibiotic coverage before drug susceptibility testing results are available, overcome multiple mechanisms of resistance, decrease risk of emergent resistance, and improve clinical outcomes (5, 6, 14). However, because it does not allow for any reductions in the dosing of a single agent, combination therapy introduces undeniable concerns about toxicities. The incidence of kidney injury with meropenem and polymyxin combination-based therapies can be up to 50% and gastrointestinal side effects up to 27% (12). Therefore, the need for novel effective agents that are also safe is of utmost importance. The incidence of ERV adverse events in our entire cohort was minimal: only one patient experienced a possible adverse event. Nevertheless, this did not lead to drug discontinuation or therapy changes and the patient achieved positive clinical outcomes. Interestingly this incidence is far less than what was reported in the clinical trials and real-world analyses, although larger studies are needed to evaluate this impact (8–11).

Our study is not without limitations. First, our inclusion criteria were conditioned on ERV utility for 72 h regardless of culture date and time. While there might be a concern for potential survival bias, it would not be appropriate to assume clinical outcomes for ERV-treated patients if the treatment period was too brief. The median duration of ERV in our cohort was ~1 week, and the median time to ERV start was timely at a median of 72 h from culture. It is well recognized that time to start effective therapy is an integral factor in clinical success, particularly in Gram-negative infections. Additionally, duration of therapy for A. baumannii regardless of infection type is typically no less than 7 days. Notably, none of the patients included in our analyses were solid organ or bone marrow transplant recipients (15). It remains unclear if this high-risk population would achieve similar clinical benefits to the population in our cohort. Furthermore, it remains a challenge to draw conclusions about toxicity and adverse events, particularly if the duration of ERV therapy was brief. Finally, MICs for ERV were reported for a few but not the majority of isolates. It is challenging to guarantee that all collaborating sites would perform ERV MIC testing, particularly at an early stage of ERV adaptation. By MIC comparison, ERV MICs are at least 2-fold lower than tigecycline MICs in A. baumannii (7). In our cohort, tigecycline testing (*n* = 17) was almost three times more common than ERV testing (*n* = 6). While this may suggest that clinicians are utilizing tigecycline susceptibility to guide ERV selection, the ability to correlate tigecycline MICs to ERV susceptibility and to clinical success remains unknown. Due to the above considerations, further studies are needed to guide ERV selection in A. baumannii, particularly when tigecycline MICs are deemed susceptible.

Given the limited therapeutic options against A. baumannii, clinicians should be encouraged to report their experience with ERV given the potential it has for this challenging pathogen. Larger, prospective studies are necessary to assess the impact of ERV in combination with other antibiotics, particularly in patients with CRAB. Finally, studies evaluating ERV versus tigecycline or other comparator agents, particularly in pneumonic and septic patients, are highly warranted.

## MATERIALS AND METHODS

Our study was a real-world, multicenter, retrospective observational evaluation at 14 geographically distinct medical centers in the United States between September 2018 and March 2021. We included patients who were (i) ≥18 years of age, (ii) had A. baumannii-positive cultures, and (iii) had been treated with ERV for ≥72 h. Common clinical comorbidities and markers of disease progression were collected. The primary outcome was 30-day mortality. Secondary outcomes included 30-day recurrence, resolution of signs and symptoms of infections, 30-day readmission, 90-day readmission, and occurrence of ERV-possible adverse effects (AE) using the common terminology criteria for adverse events (CTCAE). Resolution of signs and symptoms was defined as resolution or improvement of infection-related abnormal white blood cells/temperature or as documented by the physician in clinical notes. Thirty-day recurrence was defined as culture positive for the same organism isolated from the index culture counted 30 days from the end of ERV treatment. Combination therapy was defined as any therapy used in tandem with ERV for ≥48 h targeted for A. baumannii. Active therapy before ERV was defined as therapy deemed to have *in vitro* susceptibility for A. baumannii administered before the ERV start date. For A. baumannii, susceptibility was interpreted using the Clinical and Laboratory Standards Institute (CLSI) susceptibility breakpoints when applicable to drugs such as minocycline and tetracycline. When CLSI information was not available, the FDA antibacterial susceptibility interpretive criteria were used if available (16). Descriptive statistics were utilized for data analysis using IBM SPSS software version 27.0 (SPSS, Inc., Chicago, IL, USA).

### Ethical review.

This study design and work had been reviewed and approved by the Wayne State University Human Investigational Review Board and the DMC Research Review Committee prior to initiation. A patient consent statement was not required for this retrospective analysis.
